# Stem Cells and Labeling for Spinal Cord Injury

**DOI:** 10.3390/ijms18010006

**Published:** 2016-12-26

**Authors:** Marina Gazdic, Vladislav Volarevic, Aleksandar Arsenijevic, Slaven Erceg, Victoria Moreno-Manzano, Nebojsa Arsenijevic, Miodrag Stojkovic

**Affiliations:** 1 Center for Molecular Medicine and Stem Cell Research, Faculty of Medical Sciences, University of Kragujevac, 34000 Kragujevac, Serbia; marinagazdic87@gmail.com (M.G.); drvolarevic@yahoo.com (V.V.); aleksandar@medf.kg.ac.rs (A.A.); arne@medf.kg.ac.rs (N.A.); 2 Stem Cell Therapies in Neurodegenerative Diseases Laboratory, Centro de Investigación Príncipe Felipe, E-46012 Valencia, Spain; serceg@cipf.es; 3 National Stem Cell Bank-Valencia Node, Biomolecular and Bioinformatics Resources Platform PRB2, ISCIII, Research Center “Principe Felipe”, c/Eduardo Primo Yúfera 3, 46012 Valencia, Spain; 4 Institute of Experimental Medicine, Department of Neuroscience, Academy of Science of the Czech Republic, Videnská 1083, 142 20 Prague 4, Czech Republic; 5 Neuronal and Tissue Regeneration Laboratory, Centro de Investigación Príncipe Felipe, E-46012 Valencia, Spain; vmorenom@cipf.es; 6 Spebo Medical, 16000 Leskovac, Serbia

**Keywords:** stem cells, spinal cord injury, stem cell labeling

## Abstract

Spinal cord injury (SCI) is a devastating condition that usually results in sudden and long-lasting locomotor and sensory neuron degeneration below the lesion site. During the last two decades, the search for new therapies has been revolutionized with the improved knowledge of stem cell (SC) biology. SCs therapy offers several attractive strategies for spinal cord repair. The transplantation of SCs promotes remyelination, neurite outgrowth and axonal elongation, and activates resident or transplanted progenitor cells across the lesion cavity. However, optimized growth and differentiation protocols along with reliable safety assays should be established prior to the clinical application of SCs. Additionally, the ideal method of SCs labeling for efficient cell tracking after SCI remains a challenging issue that requires further investigation. This review summarizes the current findings on the SCs-based therapeutic strategies, and compares different SCs labeling approaches for SCI.

## 1. Introduction

**Figure 1:**
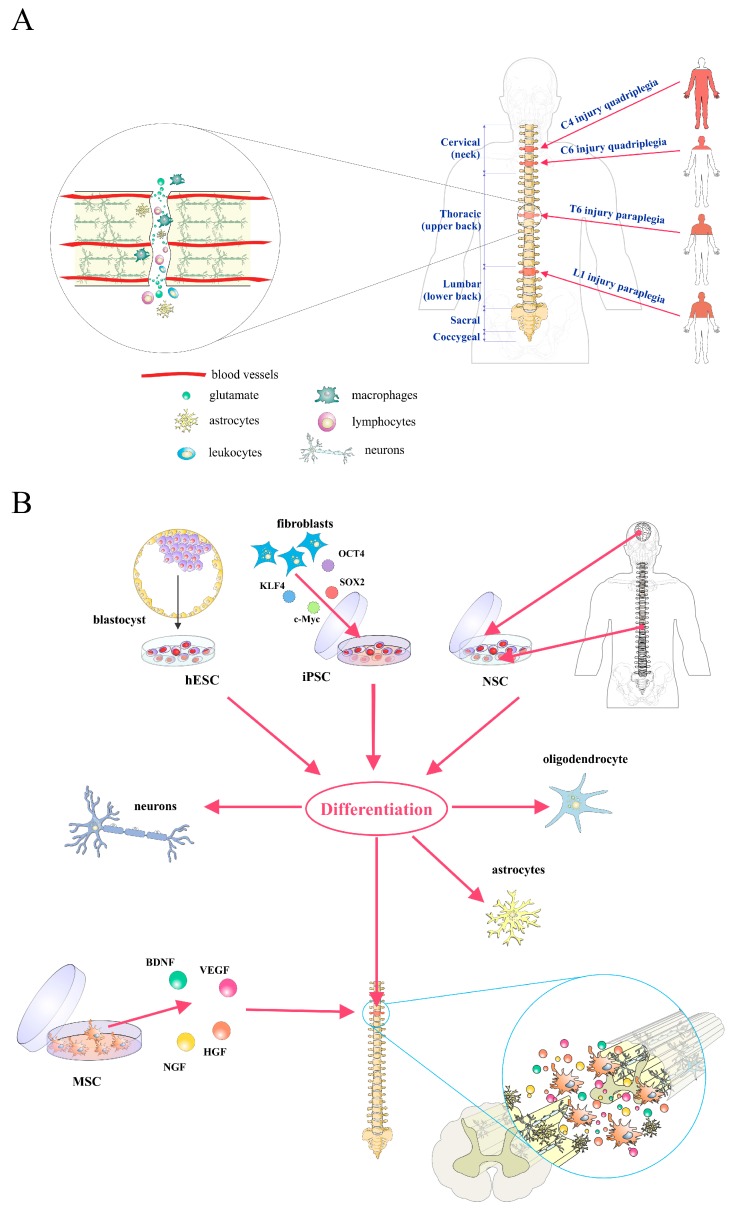
Overview of pathophysiological events and possible stem cells (SCs) treatment for spinal cord injury (SCI). (**A**) The mechanismsand clinical signs of SCI; (**B**) Potential uses of SCs as a source of neurons, oligodendrocytes, and astrocytes, as well as neuroprotectors in SCI. hESCs, human embryonic stem cells; iPSCs, induced pluripotent stem cells; NSCs, neural stem cells; MSCs, mesenchymal stem cells; BDNF, brain-derived neurotrophic factor; VEGF, vascular endothelial growth factor; NGF, nerve growth factor; HGF, hepatocyte growth factor; OCT4, octamer-binding transcription factor 4; KLF4, Kruppel-like factor 4; SOX2, sex determining region Y-box 2; c-Myc, myelocytomatosis oncogene.

Spinal cord injury (SCI) is a devastating disorder with frustrating implications for both the individual and society. Since SCI usually affects the cervical and lumbar spine, incomplete tetraplegia is currently the most frequent neurological category followed by incomplete paraplegia, complete paraplegia, and complete tetraplegia (Figure 1A) [1]. These debilitating conditions create enormous physical and emotional cost to individuals, and additionally they are significant financial burdens to the society [2]. Epidemiological data show that the incidence of SCI is approximately 54 cases per million people in the United States, or approximately 17,000 new SCI cases each year [3]. Vehicle crashes are currently the leading cause of injury followed by falls, acts of violence (primarily gunshot wounds), and sports/recreation activities, according to the National Spinal Cord Injury Statistical Center (NSCISC) [3]. Despite the progress of medical and surgical management as well as rehabilitation approaches, according to a 2016 report by the NSCISC, less than 1% of SCI patients experienced complete neurological recovery by hospital discharge. The search for new therapies has been revolutionized with the recent advances in the field of stem cell (SC) biology, which have suggested that SCs might be exploited to repair spinal cord lesions. However, there are a plethora of limitations including cell tracking and cell survival of transplanted SCs. Therefore, in this review, we address the present understanding of SCI and look at promising research avenues including SC-based treatment options for SCI. In addition, we discuss the necessity of different methods of SC labeling and imaging modalities for cell tracking and their key strengths and limitations.

## 2. Pathophysiology of Spinal Cord Injury

Understanding the pathophysiology of SCI is essential to determine the differences of potential applications of various SCs types for possible therapeutic applications after SCI. The functional loss after spinal cord trauma is due to the direct mechanical injury and consequential series of pathophysiological processes following SCI (Figure 1A, reviewed in [1]). 

The primary phase of SCI essentially involves the mechanical disruption of the normal architecture of the spinal cord, and is characterized by acute hemorrhage and ischemia [4]. The cumulative damage of neurons, astroglia, and oligodendroglia in and around the lesion site disrupts neural circuitry and leads to neurological dysfunction [5]. Acute local ischemia, electrolyte imbalance, lipid peroxidation, and glutamate accumulation further exacerbate motor, sensory, and autonomic deficits seen in patients with SCI [5,6,7].

As a consequence of blood–brain barrier damage and increased permeability, cells including neutrophils, macrophages, microglia, and T lymphocytes from the blood invade the medullar tissue, triggering an inflammatory response [1]. Massive production of free radicals, excessive release of pro-inflammatory cytokines, such as tumor necrosis factor (TNF)-α, interleukin (IL)-1β, IL-1α, IL-6, and excitatory neurotransmitters further exacerbate tissue damage [8,9]. 

In the secondary injury phase, post-traumatic necrosis and apoptosis of both functional neurons and glia including oligodendrocytes, as well as the uncontrolled form of reactive astrogliosis that occurs around the injury site, contribute greatly to the neurological dysfunction after SCI [5,10]. Weeks after injury, changes of the microenvironment associated with the neuroinflammation and cell damage trigger astrocytes proliferation in the lesion site [10]. Reactive astrocytes overexpress glial fibrillary acidic protein (GFAP), vimentin, and nestin that contribute to the formation of the glial scar, and secrete inhibitory extracellular matrix molecules such as chondroitin sulfate proteoglycans which inhibit axonal regeneration [11,12]. In spite of these negative effects of reactive astrogliosis in SCI, glial scars protect healthy neural tissue from immune cell infiltration, and re-establish physical and chemical integrity of the spinal cord [13].

## 3. Stem/Progenitor Cell Therapy for Spinal Cord Injury

Human embryonic stem cells (hESCs) are pluripotent cells, derived from the inner cell mass of the early blastocyst, that can be propagated in vitro for a long period and represent a theoretically inexhaustible source of precursor cells that could be differentiated into any cell type to study or treat human diseases [14]. Clinical applications of hESCs therapy for SCI critically depend on their ability to differentiate toward defined and purified neural cell types in vitro [1,15]. Previously, we described [14] growth conditions for the efficient and directed differentiation of hESCs toward defined neural lineages (Figure 1B). 

This in vitro system includes the use of feeder-free conditions, chemically defined medium, and the growth of differentiated hESCs without the formation of embryonal bodies [14]. The rosette-derived progenitors formed bipolar neural precursor cells (NPCs) that were positive for several markers including neuron-specific class III β-tubulin (TUJ1), Musashi, nestin, c-series ganglioside (A2B5), and microtubule-associated protein 2 (MAP2) [14]. These progenitors were able to give rise to all three major neural lineages: neurons, astrocytes, and oligodendrocytes [14]. In that study, we demonstrated that the use of extracellular inductive signals, more specifically retinoic acid, permits the efficient differentiation of hESCs into specific classes of central nervous system (CNS) neurons.

Successful clinical trials in the treatment of SCI cannot be initiated without sufficient preclinical studies using adequate animal models that closely mimic the loss of function that occurs in humans [16]. Therefore, our contribution in designing efficient protocols for differentiation of hESCs toward neurons and glia opened the possibility for testing these cells in animal models of SCI. We evaluated the therapeutic effects of transplanted hESCs-derived oligodendrocyte progenitors (OPCs) and/or motoneuron progenitors (MPs) on axonal remyelination and functional recovery of adult rats after complete spinal cord transection. Complete transection animal model of SCI causes severe behavioral (locomotor) and histological (axonal damage) changes, and has proved both useful and reliable for the investigation of the regenerative potential of different types of human cells [16]. We demonstrated that OPCs and MPs, when transplanted into adult rats after complete spinal cord transection, engrafted for at least four months, migrated at least 3 mm away from the site of injury, and had the ability to differentiate into functional oligodendrocytes and neurons significantly enhancing locomotor function [17]. About four months after transplantation of OPCs and MPs in the acute phase of SCI, enhanced astrogliosis was found, probably as a result of Notch and Janus kinase/signal transducers and activators of transcription (JAK/STAT) signaling activation. We previously showed that after SCI, these pathways were upregulated in the endogenous neural precursor cells (epSPCs) [18]. The transplanted cells in synergism with reactive astrocytes increased the expression of beneficial molecules such as nerve growth factor (NGF), laminins, fibronectin, and neurotrophins, and decreased detrimental genes such as chondroitin sulfate proteoglycan 4 (*CSPG*) and *TENASCINS*, and genes included in slit glycoprotein (SLIT)–roundabout receptor (ROBO) signaling in the lesion site resulted in less inhibitory reactive astrocytes, making them permissive to axonal growth, neuronal progenitor survival, and differentiation [13]. 

The use of hESCs was an excellent platform to introduce pluripotent stem cells as an unlimited source for targeted differentiation of hESCs and treatment of SCI. Induced pluripotent stem cells (iPSCs) have been generated from somatic cells by overexpression of several defined factors (Figure 1B) [19]. These cells and hESCs seem to be very similar in terms of morphology, cell surface marker, gene expression levels, and differentiation properties [19]. However, with iPSCs, tailored to patients for autologous use [20,21,22,23], the scientific and clinical community obtained a much better resource, avoiding at the same time immunological rejection and ethical constraints. Several studies have evaluated the efficacy of iPSCs-derived neural precursor cells (iPSC-NPs) in animal models of SCI (reviewed in [22,24]). When injected into the injured spinal cord, these cells differentiated predominantly into glia or neurons, formed synapses with host axons, and increased regeneration, leading to functional improvement [25]. Moreover, oligodendrocyte precursors can be obtained from iPSCs, which after transplantation can remyelinate host axons following SCI [26]. While some studies demonstrated the beneficial effect of iPSCs in SCI repair, Pomeshchik et al. claim that iPSCs do not promote the recovery of immunosuppressed mice with SCI [27]. This is in agreement with the finding that iPSC-NPs are able to reduce secondary damage through immunomodulation and/or neurotrophic effects [25]. 

Neural SCs (NSCs) are adult multipotent stem cells that are present in the periventricular subependymal layer and the subgranular zone of the dentate gyrus in the brain, as well as in the ependymal regions lining the central canal of the spinal cord (Figure 1B) [15]. NSCs represent an ideal candidate for SCs-based treatment of SCI based on its noticed functional improvements after transplantation with the absent of any malignant transformation offering a safety and relevance cell type for clinical applications [18]. Adult brain-derived NPCs transplanted into the injured spinal cord of rats two weeks after injury survived, integrated principally along white-matter tracts, and formed mature oligodendrocytes that repaired the myelin sheath, leading to functional and locomotor recovery [28]. However, NPCs survival was poor after transplantation into chronic lesions [28]. On the other hand, we demonstrated that acute transplantation of undifferentiated epSPCs from SCI donors, or the resulting OPCs by induced in vitro differentiation into a rat model of severe spinal cord contusion, produced a significant locomotion recovery one week after injury [18]. Transplantation of epSPCs provides trophic support and positively modulates the local immune response, reducing purinergic receptors expression associated to neurodegenerative and neuropathic pain inducing signals, thereby promoting neuronal protection and survival with axonal outgrowth [18,29]. Furthermore, we demonstrated that the capacity of activated epSPCs for functional neural regeneration is associated with reduced expression of ion channel connexin 50 at the injured and engrafted area [30] suggesting a detrimental contribution of this ion channel in regeneration of the spinal cord. On the other hand, the presence of epSPCs in the adult spinal cord suggests that endogenous SC-associated mechanisms might be exploited to repair spinal cord lesions [31]. We showed [31] that FM19G11, a new hypoxia-inducible factor (HIF) modulator, induces functional regeneration after SCI. FM19G11 confers improved self-renewal capacity of epSPCs by an early induction of a glycolytic-related response associated to a phosphatidylinositol 3-kinase (PI3K)–serine/threonine kinase (AKT)–mammalian target of rapamycin (mTOR) signaling induction [31]. Overall, FM19G11 constitutes an attractive new compound that may be potentially used to modulate SCs by its amplification or differentiation, depending on oxygen rates, for cell transplantation or endogenous activation on cell replacement therapeutical applications [31,32,33].

Due to their immunomodulatory ability and capacity for self-renewal and differentiation into tissues of mesodermal origin, mesenchymal SCs (MSCs) are adult stem cells that are most often used in preclinical and clinical studies for the treatment of various diseases including SCI. Minimal criteria for defining MSCs included plastic adherence; capability for in vitro differentiation towards osteoblasts, adipocytes, and chondroblasts; cell surface expression of cluster of differentiation (CD) 105, CD73, and CD90; and the absence of surface markers characteristic for hematopoietic cells (reviewed in [34]). Recent data suggest that plasticity, one of major characteristics of MSCs, should be extended to non-mesenchymal lineages of neuroectodermal (neurons, astrocytes, and oligodendrocytes) or endodermal (hepatocytes) origin (reviewed in [34]). However, there is a general agreement in the literature that the benefits of MSC therapy in SCI are a result of indirect environmental modification rather than direct transline age differentiation to functional oligodendrocytes or neurons [35]. In SCI treatment, MSCs are thought to act as neuroprotectors by secreting various angiogenic and neurotrophic factors such as brain-derived neurotrophic factor (BDNF), nerve growth factor (NGF), vascular endothelial growth factor (VEGF), and hepatocyte growth factor (HGF) (Figure 1B) [36], thereby providing trophic support to damaged neurons and resulting in clinical improvement in patients with SCI. Therefore, MSCs have a non-specific effect on the injured site and as such are not the best SC candidates for successful and efficient treatment of SCI.

## 4. Stem Cell Labeling and Tracking

Successful implementation of stem cell therapies for SCI requires a better understanding of cell fate after transplantation [37]. For both research and clinical purposes, tracking of SCs after their transplantation is crucial for determination of their migration, distribution, viability, and final differentiation [38]. Cell tracking can be performed by labeling cells with molecular probes that enter the cell and are trapped intracellularly (e.g., direct labeling) [37]. For direct labeling, cells are incubated with imaging probes that enter the cell via transporter uptake (i.e., 2-deoxy-2-[^18^F]fluoro-d-glucose (^18^F-FDG), 3-(2′-[^18^F]fluoroethyl)spiperone (^18^F-FESP), and 9-(4-[^18^F]fluoro-3-hydroxymethylbutyl)guanine (^18^F-FHBG)), endocytosis (i.e., superparamagnetic iron oxide nanoparticles (SPIONs), quantum dots (QDs), gold nanoparticles (AuNPs), and microbubbles), or passive diffusion (i.e., indium-111-oxine chelate (^111^In-ox)). Alternatively, cells can be labeled by overexpression of specific reporter genes that integrate into the cellular genome via viral or non-viral vectors (e.g., reporter gene labeling) [37]. Once integrated, reporter genes are transcribed into messenger RNA and translated into proteins that interact with a molecular probe for signal generation. Signals generated from cells labeled by either technique can then be visualized using imaging systems such as magnetic resonance imaging (MRI), nuclear imaging—positron emission tomography (PET)—, single photon emission computed tomography (SPECT), fluorescence imaging (FLI), or bioluminescence imaging (BLI). Here, we focus on advantages and limitations of labeling modalities that have the potential to be applied clinically.

The main limitations of direct SCs labeling are “dilution” of the intracellular markers after cell division, and the detection of probe after cell death. On the contrary, reporter genes provide long-term imaging of the progenies after labeled cell division, as well as accurate information about cell viability [39].

Among all the magnetic particles, labeling of multiple-cell lines with superparamagnetic iron-oxide (SPIO) is the most widely used and developed method [40]. The efficacy of SPIO labeling for SCs tracking after SCI has been shown in several preclinical and clinical studies [41,42,43,44,45]. For instance, Callera et al. used SPIO-labeled CD34^+^ cells injected into the spine of patients with SCI to assess their migratory capacity [41]. MRI at 20 and 35 days after cell delivery was able to detect injected cells and showed their migration towards the side of injury [41]. SPIO labeling did not affect NPCs survival, migration along white matter tracts, differentiation, and immunomodulatory property in vitro and in vivo [46]. These findings support the further use of magnetic particles in studies of NPCs transplantation in CNS diseases. A conditionally immortalized NSC line derived from human fetal spinal cord tissue (SPC-01) labeled with poly-l-lysine-coated SPIONs was implanted into the lesion one week after balloon-induced SCI [47]. Significant recovery of motor and sensory function was validated in transplanted animals two months after SCI. Grafted cells labeled with poly-l-lysine-coated SPION before transplantation were detected in the lesion on T2-weighted images (T2WI) as hypointense spots that correlated with histologic staining for iron and the human mitochondrial marker mitochondrially encoded cytochrome C oxidase II (MT-CO2) [47]. However, MRI tracking of SPIO-labeled NSCs for long-term studies may not always be reliable, and therefore should only be applied to monitor (real-time) cell delivery and initial dispersion of transplanted cells within the host tissue [48]. SPIO-labeled NSCs rapidly exocytose their iron in vivo, upon which they initiate migration. One possibility for this is that activation of the microtubule network occurswhen cells respond to migratory cues present within the host tissue. This may then simultaneously induce the active process of exocytosis, which uses the cytoskeleton framework [48].

The feasibility of in vivo tracking for MSCs labeled by SPIO with noninvasive MRI was evaluated [49,50]. Using MRI, significant reduction in signal intensity in the transplantation site was detected one and three weeks after application of SPIO-labeled MSCs [49]. Furthermore, improvement in Basso, Beattie, and Bresnahan (BBB) locomotor score, heat sensitivity, and lesion size in cell-treated animals was demonstrated [50]. All the beneficial preclinical effects of MSC therapy in SCI combined with the added value of noninvasive imaging resulted in a case report where SPIO-labeled cells were monitored with a clinical imaging system after transfer into a single SCI patient [51]. Immediately after 3 × 10^7^ SPIO-labeled MSCs intrathecal infusion, an MRI scan was performed and a hypointense signal was observed in the subarachnoid space and some in the cauda equina, but not in the cervical spine cord. Two days later a hypointense signal was observed at the injured cervical spine. There was no change in spinal cord structure based on MRI and no improvement in neurological deficit. The signal was very faint two weeks after transplantation and was not detectable at two and seven months after transplantation. In view of the fact that the number of MSCs that reach the lesioned neural tissue after administrationis considered to be low, especially at short time intervals postimplantation, the possibility of targeting MSCs labeled with SPION into the damaged spinal cord by using magnets that produce spatially modulated stray fields was studied [52,53]. Intrathecally transplanted MSCs labeled with SPION were guided by a magnetic field and successfully targeted near the lesion site in the animal spinal cord [52]. The hypointense signal was higher at the lesion site in the magnetically guided group. The higher cell migration towards the lesion site was associated with increased beneficial effects including axonal integrity and BBB locomotor rating scale [53]. However, as the signal emission by the SPIO particles depends on the density of transplanted cells, SPIO labeling is an inappropriate approach for long-term cell tracking [54]. Nevertheless, SPIO labeling suffers from common limitations to other exogenous contrasts, such as the dilution of the contrast media with cell division and the possibility that apoptotic stem cells may be phagocytized by macrophages, leading to a false positive signal on MRI [55]. 

For these reasons, radiopharmaceutical cell tracking remains an important tool for more precise evaluation of SC migration and the site of homing. Intravenous injection of 6 × 10^6^ ^111^In-ox labeled bone marrow-derived MSCs (BM-MSCs) after compressive SCI at thoracic T6–T8 level in rats led to biodistribution mainly into the spleen, liver, and kidneys, while the vertebral column showed faint migration and the spinal cord did not show any activity [56]. In contrast, when the ^111^In-ox-labeled BM-MSCs were injected into the traumatic centromedullar cavity, the gammagraphic images showed persistent homing into the lesion site, without any distribution to the rest of the organism in the 10-day imaging period [56]. These results show the utility of ^111^In labeling for being able to know the permanency and distribution of BM-MSCs after grafting procedures, and suggest the convenience of the intralesional administration of BM-MSCs, instead of the intravenous administration, in the treatment of chronic traumatic paraplegia.

**Figure 2:**
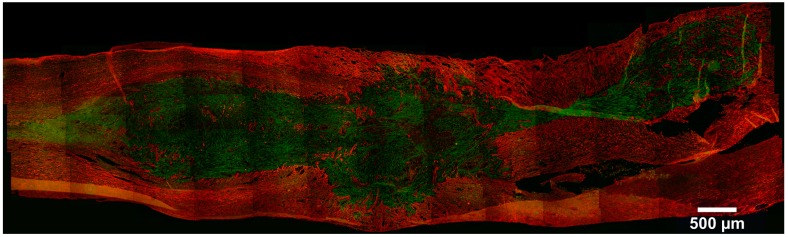
Survival and distribution of green fluorescence protein (GFP)-labeled human MSCs (**green**) in a neuron-specific class III β-tubulin (TUJ1)-labeled (**red**) longitudinal section one month after transplantation in a severe SCI rat model. Scale bar = 500 μm.

Although reporter gene imaging requires genomic manipulation and poses potential safety issues, it is the preferred labeling strategy and the most effective way to stably integrate markers into cells for both PET and BLI imaging. By inserting a gene into the cell, the marker becomes SC-specific and will not transfer to surrounding non-SCs. The use of a reporter gene allows for serial transgene expression along with key cellular properties (differentiation, proliferation, and cell viability) [57]. In vivo cell tracking by both nuclear and fluorescence imaging modalities revealed the homing of ^131^I-FIAU-labeled mouse embryo-derived NIH3T3 cells, contained dual reporter genes, herpes simplex virus type 1 thymidine kinase (HSV1-tk), and green fluorescence protein (GFP), to the SCI epicenter, and persistence three weeks after transplantation [58]. Excitation and emission wavelengths of fluorochromes have poor penetration in tissues, which limits the use of GFP-expressing stem cells for in vivo optical fluorescent imaging and potential clinical use [39]. Therefore, the most used application of fluorescent reporter genes in SC imaging is for ex vivo analysis and in histology (immunofluorescence) for post-mortem confirmation of imaging results (Figure 2). 

In contrast to FLI, where an external light source excites the fluorochrome, BLI is based on the emission of photons in reactions catalyzed by luciferase enzymes [39]. Transplanted luciferase-transduced stem or progenitor cells can be detected noninvasively in vivo by virtue of their reporter gene, which is expressed only when cells are alive [55,59]. BLI was successfully applied to investigate the optimal method of neural stem/progenitor cells (NS/PCs) transplantation to the SCI site in terms of grafted cell survival and safety. After inducing contusive SCI at the T10 level in mice, NS/PCs were transplanted into the injured animals by different procedures, including intralesional (IL), intrathecal (IT), or intravenous (IV) injection. In the IT group, the luminescence of the grafted cells, which was distributed throughout the entire subarachnoid space immediately after transplantation, was detected at the injured site one week later, and by six weeks had gradually decreased to about 0.3% of its initial level. In the IV group, no grafted cells were detected at the site of injury, but all of these mice showed luminescence in the bilateral chest, suggesting pulmonary embolism. The IL application of NS/PCs resulted in the best survival of grafted cells among the three methods investigated, and no complications were seen afterwards. Six weeks after the transplantation, BLI analysis showed that in the IL group, the luminescence intensity of the grafted cells had decreased to about 10% of its initial level, and appeared at the site of injury [60]. Additionally, luciferase-based imaging suggested that the timing of NS/PC transplantation into the injured spinal cords may be a key determinant of the fates and function of integrated cells [61]. NS/PCs expressing firefly luciferase (FLuc) reporter gene, transplanted immediately after SCI, mainly differentiated towards astrocytic glial scar tissue with only a small percentage differentiating to neurons and oligodendrocytes. In the delayed phase, however, neuronal and oligodendrocyte markers were clearly expressed, indicating the importance of the microenvironment on the differentiation of transplanted NS/PCs. Signals from these cells were detectable for up to 10 months after transplantation into the injured mouse spinal cord [61]. However, BLI is typically not as sensitive to a small number of cells as MRI or PET. This technique has been little used in humans due to concerns about the immunogenicity of the proteins and products involved. In addition, human clinical applications are limited because of the high absorbance and scattering of luminescence in living tissue. As such, BLI is likely to be limited to its current use in imaging transfected SCs in small animals, as a starting step in the development of novel imaging modalities, especially for future clinical use [57].

## 5. Conclusions

Stem cells represent the new therapeutic approach for SCI, enabling improved and efficient sensory and motor functions in animal models. Stem cells are able to promote remyelination via oligodendroglial cell replacement; produce trophic factors enhancing neurite outgrowth, axonal elongation, and fiber density; and activate resident or transplanted progenitor cells across the lesion cavity [15]. However, investigators express several concerns regarding the safety of transplantation of pluripotent SCs in SCI in humans, such as the risk of teratoma formation following pluripotent derived neural cell engraftment due to the hypothetical presence of undifferentiated SCs. The clinical use of hiPSCs circumvents the ethical issues but shares the same safety concerns as hESCs. Additionally, reprogramming methods for the generation of hiPSCs requires further improvement [62]. Also, many critical challenges remain using adult stem cells for clinical applications, including the need for pure populations of differentiated cells, inefficient tracking systems, and moderate cell survival after transplantation [1].

Due to limited information about the location and survival of transplanted cells, huge efforts are being made to improve SCs labeling and tracking methods. Every SCs tracking modality has its own advantages and disadvantages, in terms of biocompatibility of molecular probes, detection thresholds, safety, and cost-effectiveness. The use of optical imaging techniques is not feasible in humans, due to the poor tissue penetration and low resolution of signals. In contrast to optical techniques, MRI and PET/SPECT have high resolution and sensitivity in cell tracking in vivo [39]. However, MRI can not detect the number, location, and viability of cells, while PET lacks the ability of detailed anatomical imaging [63]. To create the optimal in vivo imaging modality and long-term tracking of cell fate, multimodal markers will provide the benefits of each different labeling technique. Until then, the ideal source and labeling technique of SCs for cell-based therapy for SCI remains a challenging issue that requires further investigation.
